# Genome sequencing of *Clostridium butyricum* DKU-01, isolated from infant feces

**DOI:** 10.1186/s13099-015-0055-3

**Published:** 2015-03-27

**Authors:** SangJoon Mo, Bong-Soo Kim, Sung-Jo Yun, Jung-Ju Lee, Suk-Hyun Yoon, Chung-Hun Oh

**Affiliations:** Biosafety & Validation Center, Clinical Trial Institute, Dankook University, Choenan, 330-714 Republic of Korea; Department of Life Sciences, Hallym University, Chuncheon, Gangwon-do 200-702 Republic of Korea; Department of Oral Physiology, College of Dentistry, Dankook University, Choenan, 330-714 Republic of Korea; Department of Medical Laser, Graduate School, Dankook University, Choenan, 330-714 Republic of Korea

**Keywords:** *Clostidium butyricum*, Genome sequencing, Hybrid assembly, Fructooligosaccharide

## Abstract

**Background:**

*Clostridium butyricum* is a butyric acid-producing anaerobic bacteriuma, and commonly present as gut microbiota in humans. This species has been used as a probiotic for the prevention of diarrhea in humans. In this study, we report the draft genome of *C. butyricum* DKU-01, which was isolated from infant feces, to better understand the characteristics of this strain so that it can later be used in the development of probiotic products.

**Results:**

A total of 79 contigs generated by hybrid assembly of sequences obtained from Roche 454 and Illumina Miseq sequencing systems were investigated. The assembled genome of strain DKU-01 consisted of 4,519,722 bp (28.62% G + C content) with a N_50_ contig length of 108,221 bp and 4,037 predicted CDSs. The extracted 16S rRNA gene from genome sequences of DKU-01 was similar to *Clostridium butyricum* with 99.63% pairwise similarity. The sequence of strain DKU-01 was compared with previously reported genome sequences of *C. butyricum*. The value of average nucleotide identity between strains DKU-01 and *C. butyricum* 60E3 was 98.74%, making it the most similar strain to DKU-01.

**Conclusions:**

We sequenced the DKU-01 strain isolated from infant feces, and compared it with the available genomes of *C. butyricum* on a public database. Genes related to Fructooligosaccharide utilization were detected in the genome of strain DKU-01 and compared with other genera, such as *Bifidobacterium* and *Streptococcus*. We found that strain DKU-01 can metabolize a wide range of carbohydrates in comparative genome result. Further analyses of the comparative genome and fermentation study can provide the information necessary for the development of strain DKU-01 for probiotics.

**Electronic supplementary material:**

The online version of this article (doi:10.1186/s13099-015-0055-3) contains supplementary material, which is available to authorized users.

## Background

The intestinal tract harbors various microbes that play a key role in the nutrition, health, and physiology of the host. Probiotic bacteria have been defined as live micro-organisms, when administered in sufficient amounts, which give a health benefit on the host organisms. In practice, probiotics can improve the balance of the intestinal microbiota ecosystem of the host, which is beneficial in treating variety of infant conditions including, infant diarrhea, upset stomach, eczema, and many health conditions of pre-term infants. It is also an effective alternative to antibiotics for growth promotion in a diverse number of livestock species [1]. In addition, it is reported that various immune responses are influenced by probiotics and these immunomodulatory effects have been proposed for use in several potential applications, including management of hypersensitivity reactions, prevention of infectious diarrhea, and tumor suppression [2]. A number of scientific progressions have led to the current situation, in which many probiotic strains exist and are in widespread use. For example, *Lactobacillus delbrueckii* subsp *bulgaricus*, *Lactobacillus acidophilus*, *Lactobacillus rhamnosus*, *Bifidobacterium bifidum*, *Bifidobacterium adolescentis*, *Bifidobacterium infantis*, *Streptococcus thermophilus*, *Enterococcus faecalis,* and *Enterococcus faecium* are currently being used in probiotic preparations either singly or in combination [3].

*Clostridium butyricum* is a butyric acid-producing gram-positive anaerobe which is found in soil and the intestines of healthy animals and humans, and the strain MIYAIRI 588 of *C. butyricum* has been used as a probiotic in Asia for treating and preventing non-antimicrobial induced diarrhea, antimicrobial-associated diarrhea, and constipation in humans [4,5]. In addition, *C. butyricum* produces short chain fatty acids (SCFAs), such as butyric acid, propionic acid, and acetic acid. Several reports have suggested that SCFAs have potential beneficial effects; in particular, butyric acid has a proliferative effect on intestinal mucosal cells [6] and anti-inflammatory effects [7]. Moreover, *C. butyricum* can ferment sugar and glycerol to several biofuels and precursors of biomaterials, such as H_2_, butanol, butyric acid, and 1,3-propanediol [8-11]. Due to the exhaustion of fossil fuels, the conversion of renewable biomass to valuable fuels and chemicals by microorganisms has drawn global interest [12]. For this reason, *C. butyricum* is receiving renewed attention, and it is very important to isolate the stain. Therefore, investigation of the genetic information and characteristics of *C. butyricum* DKU-01 is desired. The genome sequence analysis has been proven very useful information for understanding of *C. butyricum* DKU-01, which exhibits unique physiological and metabolic properties, and its potential metabolic engineering applications.

## Methods

### Sample collection

Fecal samples were collected from breast-feeding infants less than three months old at a hospital and postpartum care center in nearby Cheonan-Asan from 2012 to 2013. None of the infants had received antibiotics or other drugs in the months prior to sampling. Fecal samples (1 g) were collected on sterilized cotton-tipped swabs and these swabs were placed in sterile 15 ml conical tubes containing 5 ml of GYP liquid medium overlaying the top of the medium with liquid paraffin. These tubes were transferred to GasPak anaerobic jars (Becton, Dickinson and Company, NJ, USA) for further study within 30 min of the collection.

### Isolation and identification of strain

For the isolation of *C. butyricum* from infant feces, collected fecal samples were incubated at 30°C in an anaerobic chamber (Forma Anaerobic System; Thermo Fisher Scientific Inc., Waltham, MA, USA) using a gas phase of N_2_/H_2_/CO_2_ (80:10:10%, v/v) for 3 days. After enrichment, they were further serially diluted and plated on RCM (Reinforced Clostridial Medium, BD) for anaerobes in an anaerobic chamber at 30°C for 48 h. Morphologically different colonies were selected randomly. The selected colonies were purified by repeated streaking on the agar media and subcultured periodically.

All isolated strains used in this work were tested for catalase. Single colonies were selected and grown in RCM broth in order to establish pure cultures of the gas bubble-producing strains. One hundred and two isolates showing a bubble with soap skin were selected as the candidates and their biochemical profiles were confirmed using API 20A (BioMerieux, Basingstoke, UK) in order to identify the isolates. One strain, DKU-01, which showed the active formation of gas bubbles, was chosen for further studies and its identification was confirmed by 16S rRNA sequencing.

For the identification of 16S rRNA sequences, genomic DNA was extracted from the strain using the phenol extraction method. The 16S rRNA gene was amplified using a polymerase chain reaction (PCR) with the universal primers 27F (5′-AGAGTTTGATCMTGGCTCAG-3′) and 1492R (5′-TACGGTYACCTTGTTGTTACGACTT-3′). The conditions of amplification were: 94°C for 5 min denaturation, followed by 30 cycles at 94°C for 1 min, 50°C for 1 min, and 72°C for 1 min and 50 sec, with a final 4 min extension at 72°C. After amplification, the PCR products were purified using an AccuPrep PCR purification kit (BIONEER, Daejeon, Korea) and sequenced with the ABI prism™ Bigdye™ Terminator Cycle sequencing ready reaction kit in an automated ABI 3730XL capillary DNA sequencer (Applied Biosystems, Foster City, CA, USA). Strain DKU-01 was identified as *Clostridium butyricum* and was deposited in the strain collection of the Korean Culture Center of Microorganisms (Daejeon, Korea) under accession number KCTC 12367BP.

### Genome sequencing for strain DKU-01

The extracted genomic DNA was confirmed by 1% agarose gel electrophoresis and visualized under a Gel Doc system (Bio-Rad, Hercules, CA, USA) and the concentration of DNA was quantified using a PicoGreen dsDNA Assay Kit (Invitrogen, Carlsbad, CA, USA). To verify contamination of DNA or isolate culture, 16S rRNA gene (about 1.4kb) was amplified and sequenced again. After confirmation of the isolated DNA, a sequencing library for the Illumina machine was prepared using Truseq DNA LT Sample Prep Kit (Illumina, San Diego, CA, USA) and a library for the Roche 454 machine was constructed using the GS FLX Titanium Rapid Library Preparation Kit (454 Life Science, Branford, CT, USA). The genome sequencing of strain DKU-01 was performed by the Illumina Miseq (250 bp paired end sequencing) and Roche 454 (8Kb-insert paired end sequencing) sequencing systems.

### Assembly and annotation of genome sequence

The *de novo* assembly was conducted using the CLC genomic workbench 6.0 (CLC Bio, Denmark) with sequences obtained from the Miseq system and the GS Assembler 2.6 (Roche Diagnostics, Branford, CT, USA) to sequences from the Roche 454 system. The hybrid assembly of generating contigs from both systems was performed using the CodonCode Aligner (CodonCode Co. Dedham, MA, USA). Gene prediction was performed using Glimmer 3 [13], and the annotations were conducted by a homology search against Clusters of Orthologous Groups (COG) and the SEED database [14,15]. The comparative analyses of gene contents were performed using the RAST server [16]. The chromosomal region focusing fructooligosaccharide utilization in contig number 22 of strain DKU-01 was compared with synteny in similar organism using browse genome on the RAST server. The nucleotide similarities of *msm* operon were obtained by webcat (www.webact.org/WebACT/home) using BLASTN.

## Quality assurance

The potential contamination was confirmed by identification of 16S rRNA gene and comparison of the genome sequence with the published genomes of the same genus. 16S rRNA genes were extracted from assembled contigs using the rRNA selector [17] and identified by using the EzTaxon-e database (http://eztaxon-e.ezbiocloud.net) [18]. Genome sequences were compared with available genomes of *Clostridium* spp. on the NCBI database using the average nucleotide identity (ANI) values [19]. The genomic distance between genome sequences were calculated using ANI values. In a pair of genomes, the query sequence was cut into small fragments (1020bp). Then, high-scoring segment pairs between genomes were determined by the BLAST algorithm [19]. For genome tree, the calculated ANI values were converted into distances between genomes of *Clostridium*. The distance values were obtained from ANI values complement to 1. This pairwise distance matrix was used to construct a dendrogram by the Unweighted Pair Group Method with Arithmetic Mean clustering method. The digital DNA-DNA hybridization values were calculated using Genome-to-Genome Distance Calculator (GGDC; http://ggdc.dsmz.de).

## Initial findings

### General features

**Figure 1:**
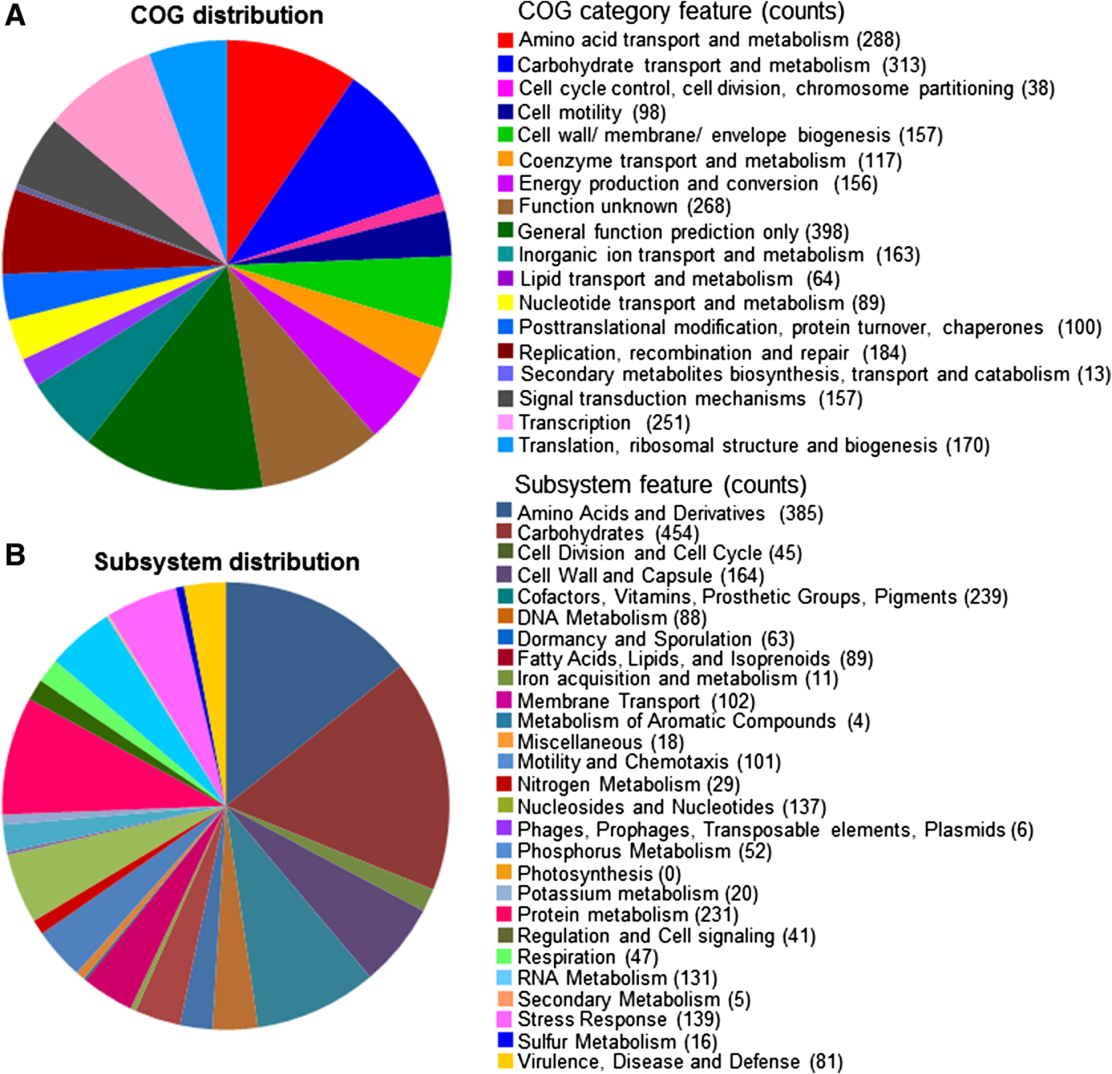
**Statistics of annotated genes for** ***Clostridium butyricum*** **DKU-01 based on COG (A) and SEED (B) databases.**

A total of 79 contigs were generated by a hybrid assembly of reads from the Illumina Miseq (12,127,925 reads of 250bp paired end; ˃554-fold coverage) and Roche 454 (319,439 reads of 8kb-insert paired end; ˃13-fold coverage) systems. The genome size of strain DKU-01 was 4,519,722 bases with 28.62% G + C content and 4,037 predicted CDSs, and 59 tRNA and 17 rRNA operons. The length of the largest contig was 334,920 bp, and the N_50_ contig was 108,221 bp. The distributions of COG and SEED subsystems of strain DKU-01 are depicted in Figure 1. Among the COG category, R (general function prediction only; 399 ORFs) and G (carbohydrate transport and metabolism; 313 ORFs) were abundant, followed by the E category (amino acid transport and metabolism; 288 ORFs), S category (function unknown; 268 ORFs) and K category (transcription; 251 ORFs). In the subsystem distribution, the subsystems of carbohydrates (454 ORFs) and amino acids and derivatives (385 ORFs) were well-represented. The subsystems of di- and oligosaccharides (127 ORFs) and central carbohydrate metabolism (91 ORFs) were prominently detected within the carbohydrate subsystem. Beta-glucoside metabolism (40 ORFs), maltose and maltodextrin utilization (38 ORFs), lactose and galactose uptake and utilization (22 ORFs), and fructooligosaccharides and raffinose utilization (15 ORFs) were abundant categories within the subsystem of di- and oligosaccharides. Pentose phosphate pathway (20 ORFs), glycolysis and gluconeogenesis (16 ORFs), and the entner-Doudoroff pathway (13 ORFs) were prominently detected within the central carbohydrate metabolism subsystem.

### Comparative analysis of DKU-01 genome with genomes of neighbor strains

**Figure 2:**
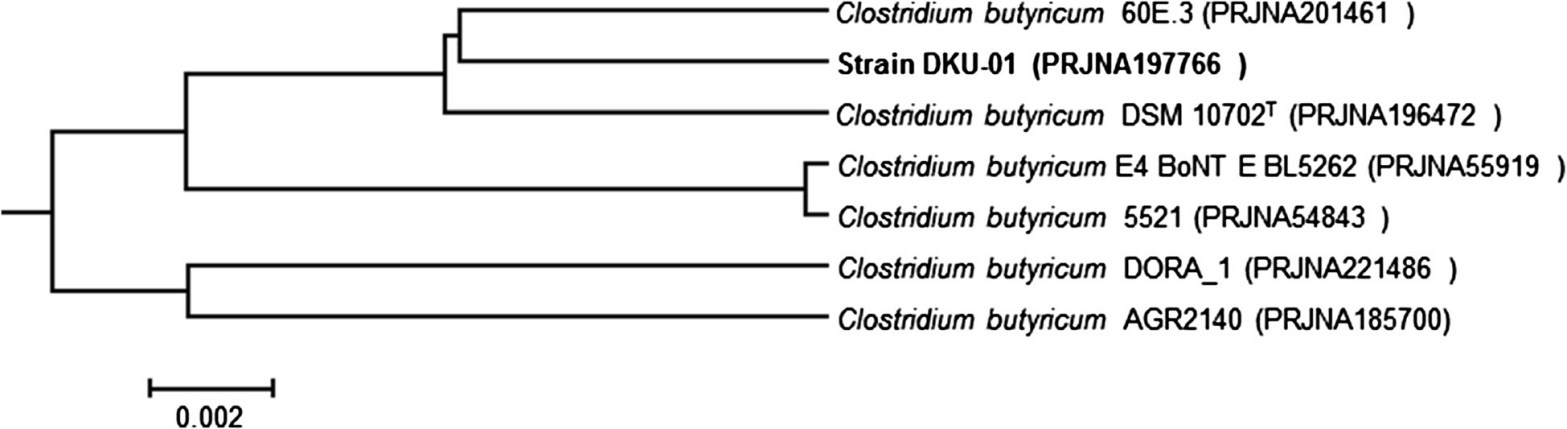
**Genome tree of strain DKU-01 with** ***Clostridium butyricum*** **strains obtained from NCBI database.**

Extracted 16S rRNA gene of strain DKU-01 had the highest pairwise similarity with *Clostridium butyricum* DSM 10702^T^ (99.63%). The genome tree of strain DKU-01 was generated by the ANI values of *C. butyricum* genomes, which are available on a public database (Figure 2). The results of 16S rRNA gene identification and genome tree indicated that the genome sequences of strain DKU-01 was free of contaminations. Six genome sequences of *C. butyricum* were compared with the genome sequences of DKU-01, and two strains of *C. butyricum,* 60E3 and DSM 10702^T^, were clustered with strain DKU-01. The ANI value of strain DKU-01 to 60E3 was 98.74% and to DSM 10702^T^ was 98.73%. The estimated DNA-DNA hybridization values of strain DHU-01 to 60E3 (90.9 ± 2%) and to DSM 10702^T^ (90.3 ± 2.08%) were obtained by the GGDC. The genomic features and subsystem distributions were compared among three strains (Additional file 1: Table S1). The lowest number of contigs was obtained from strain 60E3 (10), while the highest number of contigs was obtained from strain DSM 10702^T^ (207). The abundances of gene contents in the subsystems were similar in all three genomes.

**Figure 3:**
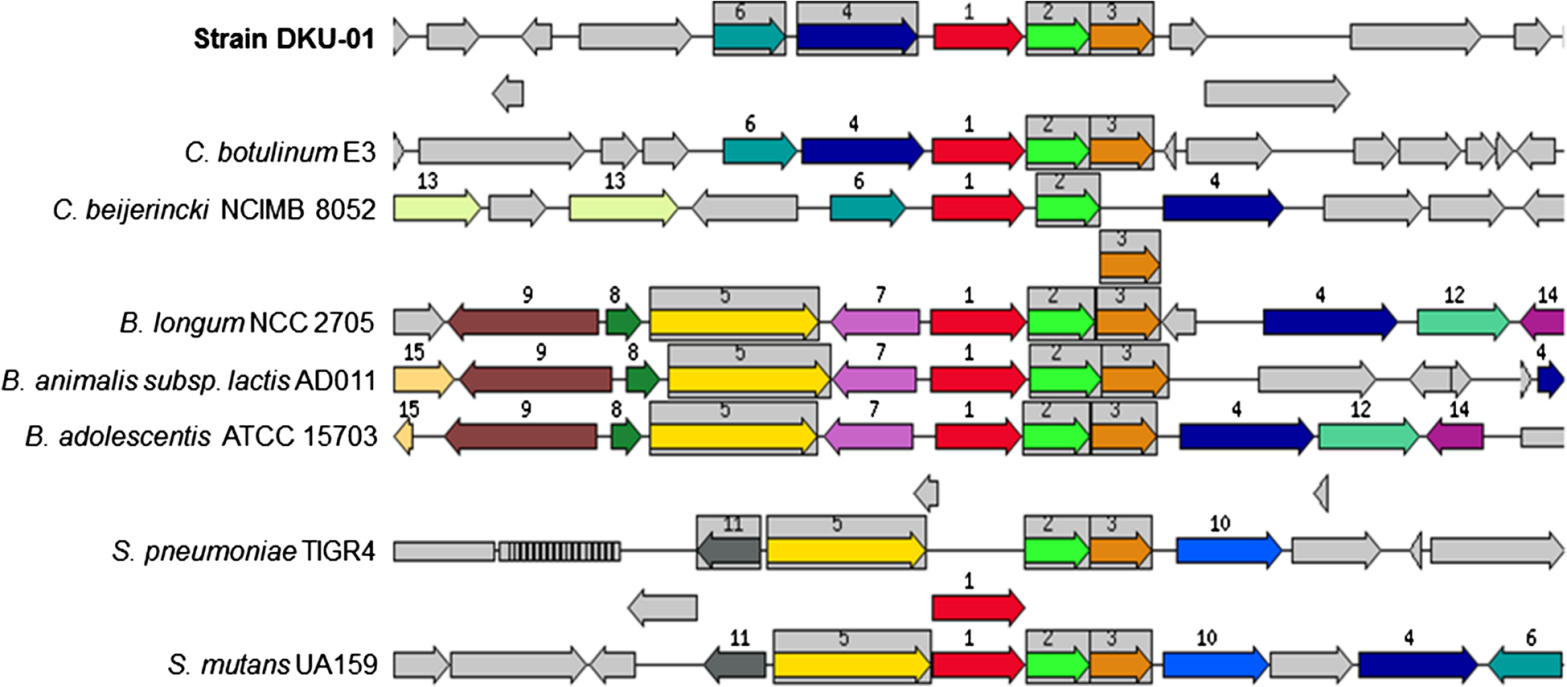
**Comparison of the** ***msm*** **cluster in contig number 22 of DKU-01 genome with selected bacteria (based on RAST annotation server).** The red arrow (number 1) indicates *MsmE*; yellow-green arrow (number 2) *MsmF*; brown arrow (number 3) *MsmG*; blue arrow (number 4) Oligo-1,6-glucosidase; yellow arrow (number 5) Alpha-galactosidase; blue-green (number 6) Catabolite control protein A; pink arrow (number 7) possible *NagC/XylR*-type transcriptional regulator; green arrow (number 8) tRNA-specific adenosine-34 deaminase; dark brown arrow (number 9) probable *NhaP*-type Na(+)/H(+) exchanger; sky-blue arrow (number 10) Sucrose phosphorylase; dark-gray arrow (number 11) *MSM* operon regulatory protein; pistachio arrow (number 12) Threonine dehydratase; lime-green arrow (number 13) Aldehyde dehydrogenase B; purple arrow (number 14) NAD-dependent protein deacetylase of SIR2 family; primrose arrow (number 15) possible phospholipase. The same number and color in the figure are sets of genes with similar sequences. Genes whose relative position is conserved in at least four other species are functionally coupled and shared gray background boxes.

We focused on fructooligosaccharide (FOS) utilization within the carbohydrate subsystem, because fructooligosaccharide has been used as a prebiotic in food products and infant formulas [20]. FOS can be a derivative of simple fructose polymers of fructose moieties attached to a sucrose molecule, and it can be degraded by a variety of lactic acid bacteria in the gut [21,22]. *Lactobacillus acidophilus* and *Bifidobacterium* spp. have been studied for the utilization of FOS [22,23], and the utilization of FOS by *Clostridium* spp. has also been reported [24]. Ten ORFs in the genome of strain DKU-01 were annotated to genes related to FOS utilization (Table 1). The chromosomal region including *MsmE*, *MsmF*, and *MsmG* in contig number 22 of strain DKU-01 was compared with synteny in *C. botulinum* E3, *C. beijerincki* NCIMB 8052, *Bifidobacterium longum* NCC 2705, *B. animalis* subsp. *lactis* AD011, *B. adolescentis* ATCC 15703, *Streptococcus pneumoniae* TIGR 4, and *S. mutans* UA159 (Figure 3). Functional analysis of the FOS gene cluster indicated that the ingestion of an oligosaccharide was interceded by an ATP-dependent binding cassette (ABC)-type transport system. Genes encoding the ABC transport system (*MsmEFGK*) as well as a putative intracellular fructosidase (*bfrA*) were found to be located in a multiple-sugar metabolism (*msm*) operon. Although the gene contents surrounding *MsmE*, *MsmF*, and *MsmG* were different between genera, the *Msm* cluster revealed a high degree of synteny. The divergence of *msm* operon sequences were analyzed by artemis comparison at nucleotide level. This highest nucleotide similarity value was investigated in *MsmF* gene from DKU-01 to *C. botulinum* E3 (85%). The similarity value of msm operon nucleotides from DKU-01 to *C. botulinum* E3 was 81.6%, and to *C. beijerincki* NCIMB 8052 was 68%. The nucleotide similarity values of other strains in Figure 3 ranged from 45.18% to 65%. The general structure of the identified gene clusters encoding upregulated genes involved in carbohydrate ingestion and catabolism indicates that typically, a three component system consisting of a regulator, transporter, and glycoside hydrolase(s) can be sufficient for utilization of potential prebiotics, irrespective of the type of transporter identified (phosphoenolpyruvate-dependent sugar phosphotransferase systems (PTS), galactoside pentose hexuronide (GPH) permease, and ABC-type transporters). Most notably, PTS permeases had higher selectivity towards disaccharides e.g. sucrose, lactose, and maltose, whereas ABC and GPH permeases showed to be induced by the longer oligosaccharides e.g. stachyose and β-galactooligosaccharides. Furthermore, similar upregulation patterns of gene expression by widely different prebiotics was interesting, remarkably the FOS-ABC transporter that was also induced by the mixed linkage polydextrose. This suggests that transporters either possess more than one specificity or less stringent molecular recognition of substrates, indicating that a wide range of carbohydrates can be metabolized by *C. butyricum* DKU-01, and likely similar commensal and probiotic bacteria.

**Table 1:** **Summary of ORFs annotated to Fructooligosaccharide utilization**

**Contig number**	**Length (bp)**	**Gene**	**Seed function**
10	1,287	*MsmE*	Multiple sugar ABC transporter, substrate-binding protein
12	1,416	*GtfA*	Sucrose phosphorylase (EC 2.4.1.7)
12	870	*MsmR*	MSM (multiple sugar metabolism) operon regulatory protein
22	1,194	*MsmE*	Multiple sugar ABC transporter, substrate-binding protein
22	864	*MsmF*	Multiple sugar ABC transporter, membrane-spanning permease protein MsmF
22	867	*MsmG*	Multiple sugar ABC transporter, membrane-spanning permease protein MsmG
36	1,110	*MsmK*	Multiple sugar ABC transporter, ATP-binding protein
36	918	*MsmR*	MSM (multiple sugar metabolism) operon regulatory protein
65	1,113	*MsmK*	Multiple sugar ABC transporter, ATP-binding protein
65	906	*MsmF*	Multiple sugar ABC transporter, membrane-spanning permease protein MsmF

## Future directions

Further analyses of the genome comparisons and FOS fermentation will provide greater information to improve the probiotic effects of strain DKU-01. This information can help to develop improved probiotics for public health.

## Availability of supporting data

The obtained genome sequence of *Clostridium butyricum* DKU-01 was deposited in the DDBJ/EMBL/GenBank under the accession APKZ00000000. The version described in this paper is the first version, APKZ01000000.

## Additional file

Additional file 1: Table S1. Comparison of genomic features and subsystem of strain DKU-01 with strain 6E3 and DSM 10702^T^.
